# Potential of La-Doped SrTiO_3_ Thin Films Grown by Metal–Organic Vapor Phase Epitaxy for Thermoelectric Applications

**DOI:** 10.1021/acs.cgd.2c01438

**Published:** 2023-03-16

**Authors:** Aykut Baki, Mohamed Abdeldayem, Carlos Morales, Jan Ingo Flege, Detlef Klimm, Oliver Bierwagen, Jutta Schwarzkopf

**Affiliations:** † Leibniz-Institut für Kristallzüchtung, Max-Born-Straße 2, 12489 Berlin, Germany; ‡ Brandenburgische Technische Universität Cottbus-Senftenberg, FG Angewandte Physik und Halbleiterspektroskopie, Konrad-Zuse-Straße 1, 03046 Cottbus, Germany; § Paul-Drude-Institut für Festkörperelektronik, Hausvogteiplatz 5-7, 10117 Berlin, Germany

## Abstract

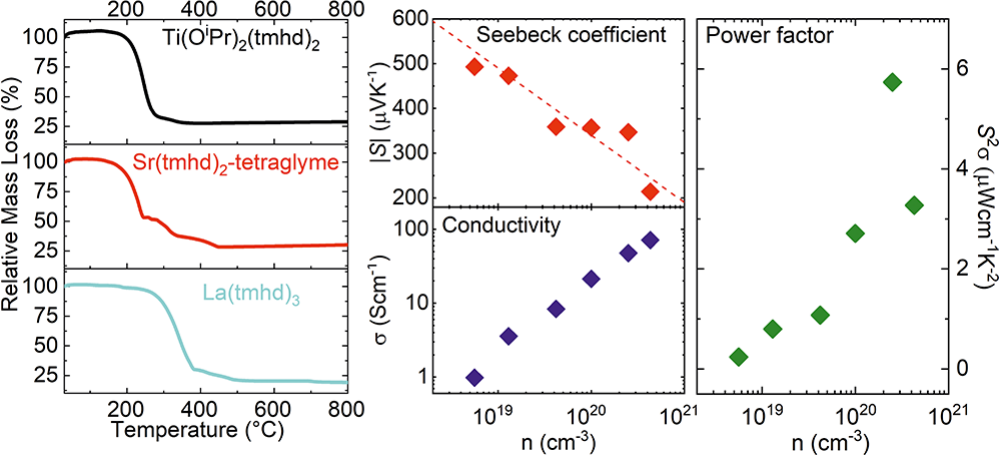

La-doped SrTiO_3_ thin films with high structural quality were homoepitaxially grown by the metal–organic vapor phase epitaxy (MOVPE) technique. Thermogravimetric characterization of the metal–organic precursors determines suitable flash evaporator temperatures for transferring the liquid source materials in the gas phase of the reactor chamber. An adjustment of the charge carrier concentration in the films, which is necessary for optimizing the thermoelectric power factor, was performed by introducing a defined amount of the metal–organic compound La(tmhd)_3_ and tetraglyme to the liquid precursor solution. X-ray diffraction and atomic force microscopy verified the occurrence of the pure perovskite phase exhibiting a high structural quality for all La concentrations. The electrical conductivity of the films obtained from Hall-effect measurements increases linearly with the La concentration in the gas phase, which is attributed to the incorporation of La^3+^ ions on the Sr^2+^ perovskite sites by substitution inferred from photoemission spectroscopy. The resulting structural defects were discussed concerning the formation of occasional Ruddlesden–Popper-like defects. The thermoelectric properties determined by Seebeck measurements demonstrate the high potential of SrTiO_3_ thin films grown by MOVPE for thermoelectric applications.

## Abstract 2

Due to its large Seebeck coefficient and chemical stability even at high temperatures, SrTiO_3_ shows great promises as environmentally friendly material for thermoelectric applications. The paper describes the homoepitaxial growth of La-doped SrTiO_3_ thin films with the metal-organic vapor phase epitaxy technique. Careful analysis of the of the MO precursors and growth conditions provides stoichiometric films with well controlled La doping concentration, high structural quality, and promising thermoelectric properties.

## Introduction

Since heat sources are found nearly everywhere, conversion of waste heat energy into electrical energy by the exploitation of the thermoelectric effect in solids promises to make a great contribution to energy harvesting concepts. Additionally, it enables the battery-independent energy supply of sensor and monitoring devices, especially interesting for health applications. However, most thermoelectric materials used are based on compounds that still contain Pb or Te.^1,2^ For sustainability reasons, there is a need for new, more environmentally friendly materials that are more stable at high temperatures and in harsh atmospheres with at least comparable and appropriate thermoelectric properties. Furthermore, a broad and versatile application of these materials requires the scale-up of the material synthesis.

Thermoelectric materials are characterized by the Seebeck coefficient (or thermopower) *S*, the electrical (σ), and thermal (κ) conductivity, which together define the dimensionless figure of merit . Highly efficient materials are specified by a high power factor *S*^2^σ and low thermal conductivity κ. However, regarding the maximization of the power factor, it has to be considered that material properties are interrelated and have conflicting effects on the power factor. This relates, on the one hand, to the charge carrier concentration *n*, which has an opposite impact on the Seebeck coefficient (*S* ∼ *n*^–2/3^) and on the electrical conductivity (σ ∼ *n*).^3^ On the other hand, this concerns the charge carrier’s effective masses. Large effective masses provide high thermopower but low electrical conductivity due to low carrier mobility,^4^ which is often found in ionic materials. Therefore, optimizing the thermoelectric properties requires finding a compromise in the charge carrier density and a balance between high effective masses and high mobilities.

SrTiO_3_ is a promising material for thermoelectric applications because it exhibits a large Seebeck coefficient even at high temperatures,^5−7^ and it is chemically inert and stable up to 1000 °C. Semiconducting n-type doped SrTiO_3_ has been achieved by the incorporation of different elements like Nb^8^ or Ta^9^ on the Ti site or Y,^10^ Sm,^11^ or La^12^ on the Sr site. A weakness of SrTiO_3_ regarding its use in thermoelectric devices is the relatively low electrical conductivity,^13^ based on the high effective mass and the resulting low charge carrier mobility, and simultaneously high thermal conductivity κ (about 5–18 Wm^–1^ K^–1^),^14^ which is reasoned by the centrosymmetric cubic structure that offers low effective phonon scattering centers. This weakness can be mitigated, for instance, by manufacturing superlattice heterostructures (such as Si/Ge^15^ or PbTe/PbSe_0.20_Te_0.80_^16^ superlattices), which leads to a reduction of thermal conductivity,^17^ or by decoupling the thermal and electronic transport by a suitable choice of the film and substrate thermal conductivities and respective thicknesses.^18^

In order to achieve sufficiently high electrical conductivity, films must be grown with high crystalline perfection and precisely controlled doping concentration. This requires a deposition method that allows the epitaxial growth of films with high structural quality and low defect density. Epitaxial SrTiO_3_ films have been obtained by molecular beam epitaxy (MBE)^19,20^ and pulsed laser deposition (PLD).^21,22^ However, these methods operate at low oxygen partial pressures, often suffering from oxygen vacancies and limited upscaling potential. In contrast, metal–organic vapor phase epitaxy (MOVPE) allows for the epitaxial growth of stoichiometric films^23^ at oxygen partial pressures that are orders of magnitude higher than in MBE or PLD, and for film growth near thermodynamic equilibrium. The independent control of the partial pressure of all constituents (including the doping element) permits the precise adjustment of the chemical composition of the films. A further advantage of MOVPE is the easy upscaling possibility, which makes it suitable for industrial applications. However, up to now, very few reports have addressed the epitaxial growth of SrTiO_3_ with MOVPE,^24−26^ due to the limited availability, complex handling, and challenging delivery into the gas phase of the metal–organic (MO) precursors.

In this paper, we report the growth of La-doped SrTiO_3_ epitaxial films by MOVPE with controlled electrical properties, i.e., with tailored charge carrier concentration, conductivity, and mobility, via tuning the composition of the gas phase. We have selected La as the doping element due to the occurrence of only the trivalent state La^3+^ and the similarity between the ionic radii of La^3+^ and Sr^2+^ (1.36 and 1.44 Å, respectively). By optimizing the growth conditions and precise adjustment of the La doping concentration in the films, we reach a thermoelectric power factor of up to 5.8 μWcm^–1^ K^–2^.

## Experimental Section

La-doped SrTiO_3_ thin films with a thickness between 30 and 50 nm were grown via liquid-delivery spin MOVPE in an oxygen-argon atmosphere at a chamber pressure of 15 mbar. The metal–organic (MO) compounds Sr(tmhd)_2_-tetraglyme,^27^ Ti(O^i^P_r_)_2_(tmhd)_2_,^28^ and La(tmhd)_3_^29^ were solved in dry toluene (<2 to 3 ppm H_2_O) and used as precursors. The deposition method requires evaporating the liquid MO precursors in a flash evaporator. Since our MOVPE setup is equipped with only two independent vaporization lines, the two precursors for the A-site metal ions (La and Sr) are mixed in one container, pumped simultaneously into the same flash evaporator, and transported together with the carrier gas argon into the reactor chamber. The second precursor line was used for the Ti precursor so that A- and B-site precursors are only mixed in the gas phase of the reactor above the heated substrates. For the La-doped SrTiO_3_ thin films, the substrate temperature and the Ar/O_2_ flux ratio were adjusted to 710 °C and 1500 sccm/5000 sccm, respectively. A more detailed description of the epitaxial growth of SrTiO_3_ thin films is given in ref (1). For the deposition of the single-oxide phases SrO/La(OH)_3_, we used slightly different growth conditions with a substrate temperature of 600 °C and an Ar/O_2_ flux ratio of 2500sccm/4000sccm.

The SrTiO_3_ and SrO/La(OH)_3_ films were grown on (100)-oriented SrTiO_3_ substrates (Crystec GmbH) with 0.1° off-cut and prepared by the routine described by Kawasaki et al.^30^ This preparation leads to an atomically smooth step-and-terrace surface structure with a terrace width of about 200 nm with single TiO_2_ surface termination.

Thermogravimetry (TG) setup from NETZSCH STA 409 CD was used to investigate the pyrolysis behavior of the employed precursors. TG signals were measured under a constant heating rate of 3 K/min from room temperature to 800 °C. The applied atmosphere consisted of an O_2_–Ar mixture (with O_2_ and Ar flow rates of 30 and 70 mL min^–1^, respectively) under atmospheric pressure. The absolute temperatures were precisely calibrated by metal reference samples ensuring a temperature error bar of ±2 K.

Structural properties of the films (like phase formation, orientation, vertical lattice parameter, and film thickness) were characterized by high-resolution X-ray diffraction (HRXRD) performed in a Rigaku SmartLab diffractometer using a monochromator to select the Cu Kα_1_ line (λ = 1.54056 Å) from a well collimated primary beam (asymmetric Ge(220) 2-bounce monochromator channel-cut crystal with an incident beam divergence of 12 arc. sec) and a HyPix-3000 horizontal 2D detector. Simulations of the XRD scans were performed to evaluate film thickness and peak position by the simulation software RCRefSimW (Version 1.08).

Ex situ X-ray photoelectron spectroscopy (XPS) measurements were performed with an Omicron EA 125 hemispherical electron analyzer using Al Kα radiation to avoid the overlap of the La 3d main photoemission region and the Auger signals from Ti. The pass energy was set to 20 eV, yielding an overall spectral resolution of about 1.1 eV. Sample charging was corrected by calibrating the adventitious carbon C 1s peak to 284.8 eV.^31^ The samples were not sputtered for cleaning purposes to avoid changing the chemical state (i.e., reduction), generation of defects, or changes in Sr/Ti/La stoichiometry by preferential sputtering.^32^ The spectra were fitted using the XPSPeak software, version 4.1, including a Shirley background removal and the use of line profiles calculated from symmetric Gaussian–Lorentzian sums (70% G, 30% L). During the fitting, the spin–orbit shift and the doublet intensity ratio were kept fixed at Δ = 1.76 eV and 0.69 for Sr 3d and at Δ = 5.76 eV and 0.5 for Ti 2p, respectively. In all analyzed spectra, the full width at half-maximum was fixed at 1.3 eV for both Sr components and 1.4 and 2.2 eV for Ti 2p, taking into account the Coster–Kronig effect. The La 3d_5/2_ region was fitted applying the model for La_2_O_3_ reported by Sunding et al.^33^

Conductive thin films were investigated regarding their electrical properties by means of Hall-effect measurements using a Lake Shore HMS 7504 system in Van-der-Pauw geometry. Ohmic contacts of Ti (20 nm)/Au (80 nm) were evaporated on all corners of the 5 × 5 mm^2^ sample by electron beam evaporation in vacuum. The ohmic behavior of the contacts was tested before performing the Hall-effect measurements by current–voltage characteristics. As a reference to exclude potential parallel conductivity in the SrTiO_3_ substrate, Hg-CV measurements^34^ were performed on a 30 nm thick undoped SrTiO_3_ film as well as an as-purchased, undoped SrTiO_3_ substrate.

Thermoelectric properties of the La-doped films were investigated by Seebeck effect measurements using a custom-built setup described in Preissler et al.^35^ In the present work, we are using a modified measuring scheme (similar to that described in ref (36)), that is utilizing the same type of leg of the type-E thermocouples also to probe the thermovoltage *V*_S_ across the sample. Nanovoltmeters (Keithley K181 and Keithley K2182) were used to measure all voltages. *V*_S_ was measured at 8–10 data points covering temperature differences Δ*T* = 0–10 K. The Seebeck coefficient *S* of the sample was determined by the slope Δ*V*_S_/Δ*T* corrected by the Seebeck coefficient *S*_tc_ = −44 μV/K of the used leg of the thermocouple, i.e., *S* = Δ*V*_S_/Δ*T* + *S*_tc_.

## Results and Discussion

### Precursor Characterization

**Figure 1:**
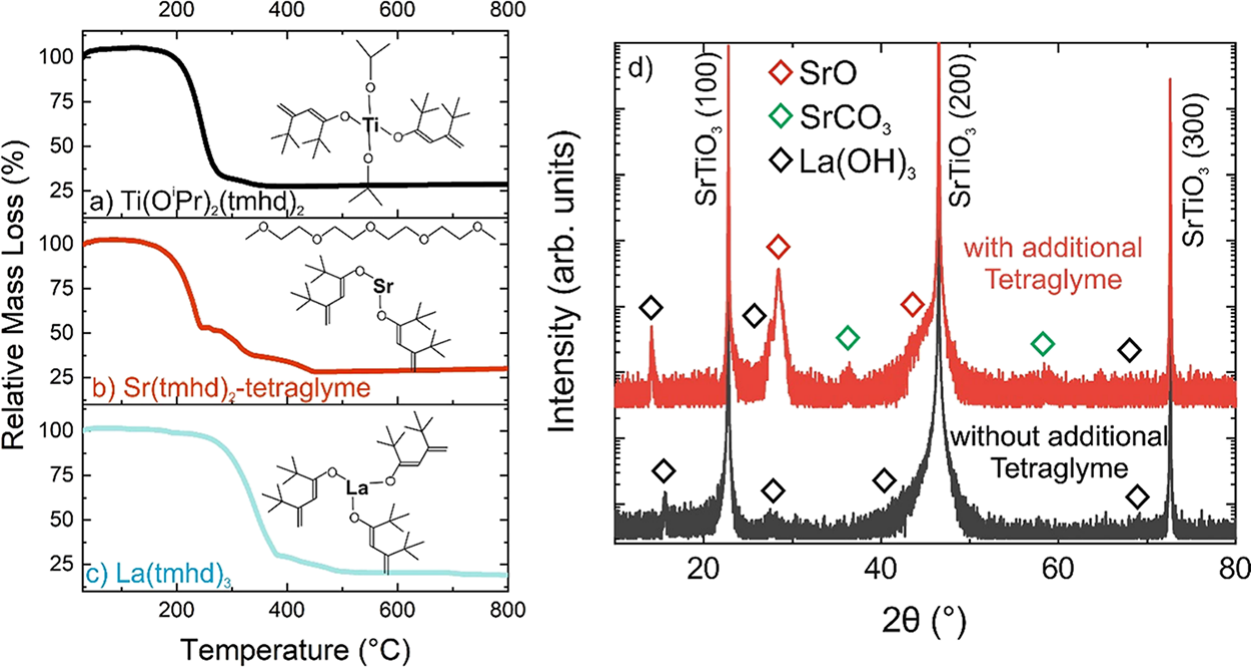
Thermogravimetry curves of the precursors (a) Ti(O^i^P_r_)_2_(tmhd)_2_ (black), (b) Sr(tmhd)_2_-tetraglyme (red), and (c) La(tmhd)_3_ (cyan). Schematic representations of the structure of the MO precursors are given in the diagrams. (d) HRXRD patterns of thin films grown on (100) SrTiO_3_ substrates by vaporizing Sr(tmhd)_2_-tetraglyme and La(tmhd)_3_ dissolved together in dry toluene in the same precursor container. Depositions were performed without extra tetraglyme (black curve) and with extra tetraglyme (red curve). The observed reflection peaks are attributed to La(OH)_3_ (black diamonds),^38^ SrO (red diamonds), and SrCO_3_ (green diamonds).^39^

Since the used MOVPE setup only allows the independent evaporation of two precursor solutions, one precursor line has to be used for two MO precursors, while the third precursor is separated from them until the toluene–argon-precursor mixtures are introduced into the reactor chamber. Due to the similarity of the MO precursors with respect to their ligands, our approach was to mix the La and Sr precursor (La(tmhd)_3_ and Sr(tmhd)_2_-tetraglyme, respectively) in one precursor container with dry toluene and to evaporate them together. Instead of a pure Sr(tmhd)_2_ precursor, we used Sr(tmhd)_2_ with the Lewis base adduct tetraglyme because it improves the thermal stability and solubility of the Sr precursor and prevents the agglomeration of Sr precursors (or in general of the alkaline-earth precursors^37^). Initially, we investigated the thermal characteristics of the precursors and verified their chemical compatibility. For this purpose, TG measurements of all three precursors were performed in the temperature range between RT and 800 °C, see Figure 1a–c.

For all three precursors, a steep drop is observed at elevated temperatures, which indicates the onset of pyrolysis related to the splitting off (detachment) of the different ligands.^27,29,40^ However, the kinks occur at different temperatures, which increases in the sequence Sr(tmhd)_2_-tetraglyme – Ti(O^i^P_r_)_2_(tmhd)_2_ – La(tmhd)_3_. Furthermore, while for the La und Ti precursors only two temperature ranges with different slopes can be identified, the TG curve of the Sr precursor obviously exhibits three temperature ranges. This indicates a binding interaction between the Sr(tmhd)_2_ and the Lewis base tetraglyme.^37^ Full decomposition is completed for the three precursors at three different temperatures.

Our intention of this work was essentially to establish suitable evaporator conditions for the precursors. Therefore, despite clear differences in the thermal behavior of the three precursors, we will not explore this in more detail within this paper but concentrate on determining a suitable evaporator temperature. Ideally, the temperature is in the range of the first slope, where effective evaporation is achieved but not yet decomposition. Even if the temperature of the two precursor lines can be set independently, a temperature of 210 °C for all three precursors is a good compromise for ensuring this condition. However, it has to be considered that the evaporation efficiency is different for all three precursors, meaning that the ratio of the chemical elements in the gas phase has to be adjusted by fine-tuning the concentration of the precursors in the toluene.

### Film Growth

With this temperature setting of the flash evaporators, in the first step, films were grown at 600 °C substrate temperature only with the precursors La(tmhd)_3_ and Sr(tmhd)_2_-tetraglyme dissolved in dry toluene in a single precursor container. We used an atomic concentration ratio of La/Sr = 1:1. The HRXRD pattern of the resulting film in Figure 1d (black curve) reveals only a La(OH)_3_ phase;^38^ no Sr-containing phases can be detected in the film. We assume that due to the higher electrophilic property of La(tmd)_3_ compared to Sr(tmhd)_2_, the polar tetraglyme, which is only weakly bonded to the Sr(tmhd)_2_, tends to attach preferentially to the La(tmhd)_3_ molecule. Consequently, the twofold positive charge of the large Sr ion (ionic radius Sr^2+^: 1.44 Å) is no longer shielded by the tetraglyme. Hence, solubility in toluene remarkably decreases, and Sr(tmhd)_2_ molecules are less protected against aggregation.^41^ This corresponds to our observation that a pure Sr(tmhd)_2_ compound can rarely be solved even in dry toluene (<2 to 3 ppm H_2_O).

In order to avoid the detachment of the tetraglyme molecule from the Sr(tmhd)_2_ precursor, we additionally introduced one tetraglyme molecule per La(tmhd)_3_ precursor molecule into the solution. Subsequently, the same deposition run was performed, again with an atomic concentration ratio of La/Sr = 1:1. The corresponding XRD pattern of the film verifies the occurrence of both La and Sr^39^ containing phases, see Figure 1d (red curve), which indicates that both precursors La(tmhd)_3_ and Sr(tmhd)_2_ were successfully transferred into the gas phase of the reactor chamber. However, it should be pointed out that the peak intensities of the La(OH)_3_ phase are low for both depositions with and without extra tetraglyme (Figure 1d). This is attributed to the lower evaporation efficiency of the La(tmhd)_3_ compared to Sr(tmhd)_2_ at 210 °C, see Figure 1b,c.

From our results so far, we conclude that Sr and La can be introduced into the gas phase and incorporated into the growing film from a single precursor solution when additional tetraglyme is used to stabilize the Sr precursor and the low efficiency of the La(tmhd)_3_ evaporation at 210 °C is considered. Therefore, in the following, we investigate the influence of the La(tmhd)_3_ concentration on the SrTiO_3_ film properties by systematically varying the La/Sr concentration ratio in the precursor solution. Since the evaporation efficiencies of the three precursors in the flash evaporators are unknown, we use the concentrations (molarities) of the La, Sr, and Ti in toluene as parameters. While the Sr and Ti concentrations were kept constant (4 and 7.5 mM, respectively), the La concentration varied between 0.00 mM (undoped film) and 2.30 mM.

### Structural Characterization

**Figure 2:**
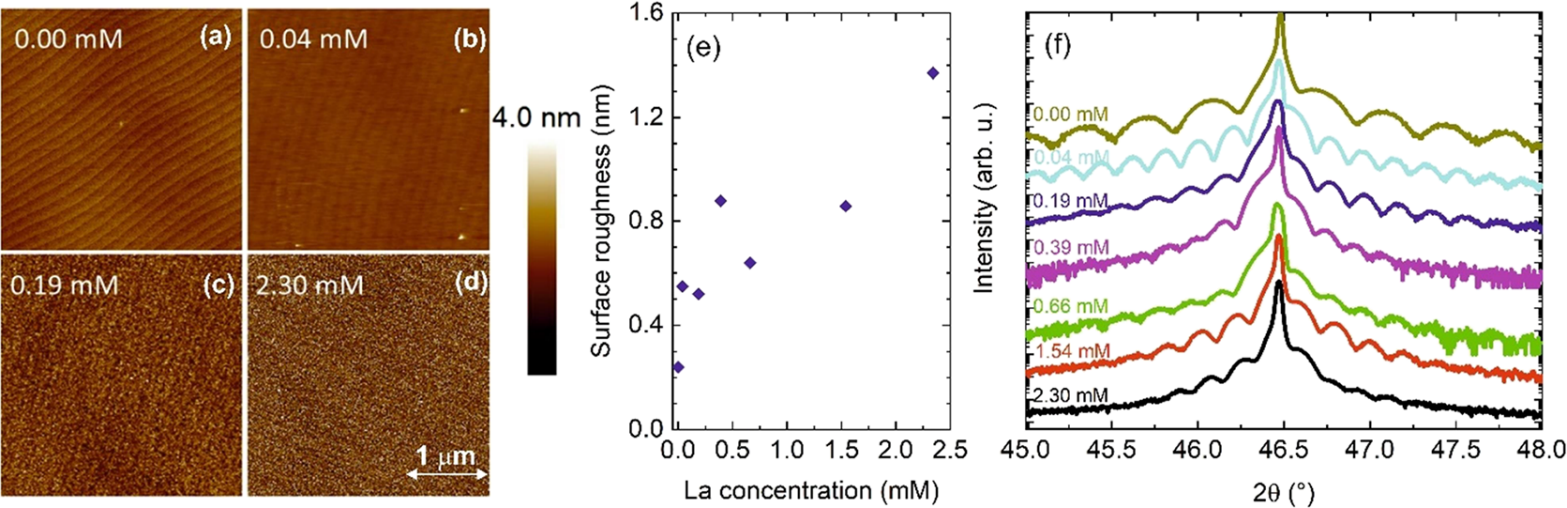
(a–d) AFM images (scan size 4 × 4 μm^2^) of the surface morphology of the undoped SrTiO_3_ thin film, (a) c(La) = 0.00 mM, and exemplarily for La-doped SrTiO_3_ thin films with La-concentrations (b) 0.04 mM, (c) 0.19 mM, and (d) 2.30 mM. (e) Surface roughness (rms) as a function of the La concentration. (f) θ/2θ HRXRD scans in the 2θ range between 45 and 48° of 30–50 nm thick La-doped SrTiO_3_ films homoepitaxially grown on a (100) SrTiO_3_ substrate with a La concentration between 0.00 mM (undoped) and 2.30 mM.

The AFM image of the undoped SrTiO_3_ thin film (c(La) = 0.00 mM, see Figure 2a) shows an atomically smooth step-and-terrace surface structure, which corresponds to the stepped surface prior to growth due to the 0.1° off-orientation of the SrTiO_3_ substrate. With the introduction of the La precursor into the gas phase, the growth mode changes from step-flow to the 3D growth mode of coalesced islands (Figure 2b–d). With increasing La concentration, the island density on the terrace increases, which results in a continuous increase of the surface roughness (rms) from 0.3 nm (undoped film) to 1.3 nm for the highest La concentration (Figure 2e).

In long-range θ/2θ HRXRD scans between 10 and 80° (see Figure S1a in the Supporting Information), only the (100) Bragg reflection peak and higher orders caused by the SrTiO_3_ substrate and film are observed. Neither a foreign phase nor a different surface orientation could be detected. Measurements in the vicinity of the (200) SrTiO_3_ substrate peak are presented with higher magnification in Figure 2f for the undoped and the La-doped SrTiO_3_ thin films. All HRXRD patterns exhibit a distinct SrTiO_3_ substrate peak and additionally a film contribution only visible as a shoulder at the substrate peak. Pronounced oscillations are visible on both sides of the substrate and film Bragg peaks, which indicate high structural quality as well as smooth surfaces and interfaces of the films. The experimental XRD data were evaluated by the simulation software RCRefSimW (Version 1.08) based on the assumption of a homogeneous fully strained layer on the SrTiO_3_ substrate. Coherent film growth was verified by reciprocal space maps in the vicinity of the asymmetric (204) Bragg reflection for the undoped as well as the different La concentrations (see the Supporting Information, Figure S2). However, a satisfactory match between the experiment and simulation concerning the angular position of the film contribution and the thickness fringes could only be achieved by a single film component in the case of the undoped layer (see the Supporting Information, Figure S3a). For the La-doped films, at least two or three sublayers with different vertical lattice parameters or Debye–Waller factors were required to obtain a reasonably decent match (see the Supporting Information, Figure S3b–g).

We conclude from the simulations that only the undoped layer is homogeneous, while the doped layers show a slight inhomogeneity with the film depth, similar to what we observed for intentional off-stoichiometric, Sr-deficient SrTiO_3_ thin films.^23^ This is also supported by the less pronounced and slightly irregular thickness oscillations, especially for the films with higher La doping.

Furthermore, we found a vertical lattice parameter of (3.909 ± 0.001) Å for the undoped SrTiO_3_ thin film. This value is slightly larger than the lattice parameter of unstrained, stoichiometric SrTiO_3_ and indicates a small off-stoichiometry in the film. For the La-doped films, the vertical lattice parameters are typically in the range of (3.911 ± 0.001) Å, which verifies a minimal but measurable increase of the lattice dimensions.

This increase in vertical lattice parameters can be caused on the one hand by an off-stoichiometric composition. However, post-growth annealing of the films in pure oxygen flow at 800 °C for 1 h does not lead to any significant change in the XRD pattern (see Figure S1a,b in the Supporting Information). That means an off-stoichiometry is not due to an oxygen deficiency as it is often observed in films grown by PLD or MBE,^21^ but possibly to a slightly enhanced Sr deficiency.^23^ On the other hand, LaTiO_3_ (3.928 Å)^42^ exhibits a larger lattice parameter than SrTiO_3_ (3.905 Å),^43^ which might lead to an enlarged size of the unit cell according to Vegard’s law, if La concentration is high.

### Chemical Properties

Ex situ XPS measurements were carried out on three selected films with different La concentrations (0.03, 0.05, and 0.17 mM) to determine the trends in the metal ions ratio composition (Sr/Ti/La) and their respective oxidation states. Table 1 shows the atomic percentage (atom %) of Ti, Sr, and La, obtained after background removal and considering the total raw area weighted by the corresponding atomic sensitivity factor. Despite the relatively high uncertainty (∼10%) of this kind of quantitative XPS measurements for elements with atomic concentrations below 1–2%, i.e., for the La dopant, we see a robust trend in the relative Sr/Ti/La concentrations, with the atomic fraction of Ti remaining constant and the Sr content decreasing as the amount of La incorporated into the SrTiO_3_ films is increased. These results indicate that La ions substitute the Sr in the perovskite lattice, which is supported by the similar ionic radii of La^3+^ and Sr^2+^ (1.36 and 1.44 Å, respectively, for 12-fold coordination) compared to Ti^4+^ (0.65 Å). Similar conclusions have previously been reported elsewhere.^44,45^

**Table 1:** Atomic Percentage of Ti, Sr, and La in Three SrTiO_3_ Films Grown with Different La Concentrations in the Source Solutions

concentration of La precursor in source solution (mM)	Ti (atom %)	Sr (atom %)	La (atom %)
0.03	49.8	50.0	0.2
0.05	50.3	48.7	1.0
0.17	50.0	48.3	1.7

**Figure 3:**
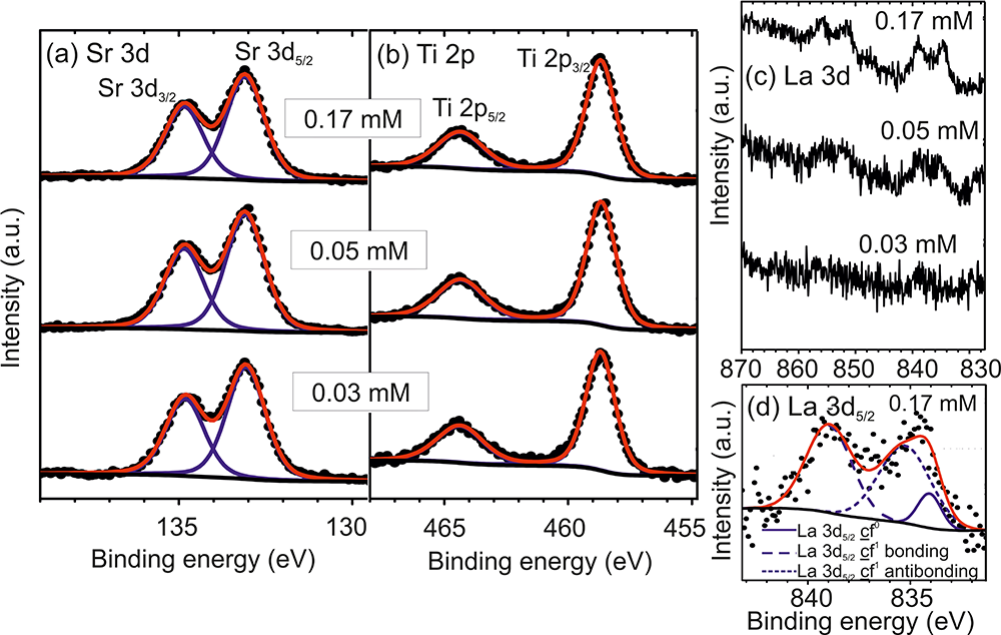
Measured XPS core level spectra (black) of (a) Sr 3d, (b) Ti 2p, and (c) La 3d for three different La concentrations 0.03, 0.05, and 0.17 mM. (d) Higher magnification of the La 3d_5/2_ core level spectra and corresponding fit for the 0.17 mM La sample. In (a), (b), and (d) simulation data are included, where the blue curves refer to the single components of the Sr 3d or La 3d doublet and the red curves to the envelopes.

The Sr 3d, Ti 2p, and La 3d regions are shown in Figure 3a–c, respectively. As indicated in the Experimental Section, the three samples have consistently been fitted using the same set of parameters defining the lineshapes, leading to the expected fit for the SrTiO_3_ matrix in all cases. The Sr 3d corresponds to the expected Sr^2+^ state without the presence of different surface Sr-based species, such as SrO or SrCO_3_. Moreover, the Ti 2p spectra reveal a single contribution associated with the Ti^4+^ oxidation state, different from what was previously reported for highly doped SrTiO_3_ films with Nb and La, where a well-defined Ti^3+^ shoulder appeared.^46,47^ Although possible vias for charge compensation due to La doping will be discussed later considering also the thermoelectric characterization, the absence of Ti^3+^ states in our fit could in principle be related to the low La concentration, the relatively low energy resolution of our XPS analyzer, and the high oxygen dose compared to the growth methods used in the aforementioned studies. These results are in line with our HRXRD measurements, where neither foreign phases nor different crystalline orientations could be detected. In the case of La, the signal-to-noise ratio is limited by the low concentrations. Nevertheless, we report similar spectra exhibiting the characteristic multiplet splitting of the two spin–orbit split components La 3d_3/2_ and La 3d_5/2_ for the three samples, especially for La concentrations of 0.05 and 0.17 mM (0.03 mM sample is in the detection limit), which are also very similar to what has been previously reported in La-doped SrTiO_3_ films^44^ and La_2_O_3_ films on silicon.^48^ Moreover, Figure 3d shows the La 3d_5/2_ fit following the model reported by Sunding et al. for La_2_O_3,_^33^ also supporting the incorporation of lanthanum with La^3+^ oxidation state. Besides, the presence of La(OH)_3_ phases on the surface could be discarded in two ways. First, the evolution of the Sr/Ti/La atomic ratios with the incorporation of La does not correspond to a direct excess of La, but with a gradual decrease of Sr, while Ti remains constant. Second, the spectrum of La(OH)_3_ is different from that of La_2_O_3._^33^ Even though La/SrTiO_3_ films may show a slightly different La 3d_5/2_ spectrum compared to pure La_2_O_3_, the applicability of the latter fitting model still confirms the absence of La(OH)_3_ species.

### (Thermo-) Electrical Measurements

**Figure 4:**
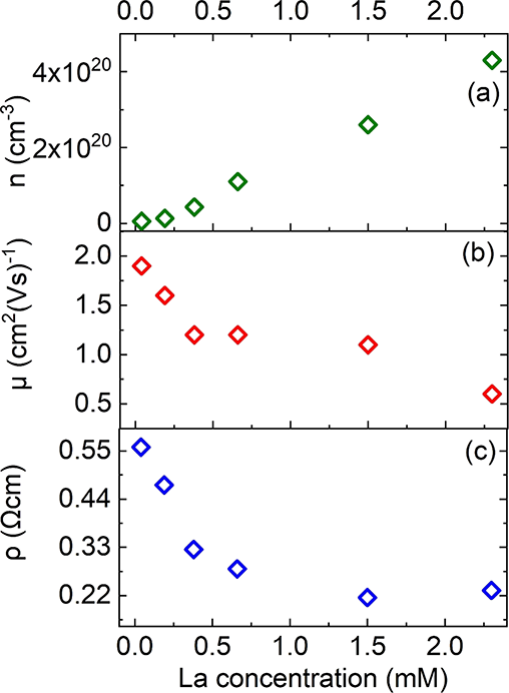
(a) Charge carrier density *n*, (b) mobility μ, and (c) resistivity ρ as a function of the La concentration determined by Hall-effect measurements.

In order to calculate the power factor *S*^2^σ, both Seebeck coefficient *S* and electrical conductivity σ have been determined. First, we consider the electrical properties obtained from Hall-effect measurements. From our Hg-CV measurements of an undoped SrTiO_3_ thin film and undoped SrTiO_3_ substrate (for more details see the Supporting Information), we exclude a parallel electrical conductivity in the SrTiO_3_ substrate that would influence the transport properties of the La-doped films. Charge carrier density *n*, charge carrier mobility μ, and resistivity ρ of the SrTiO_3_ thin films as a function of the La concentration in the precursor solution are shown in Figure 4a–c, respectively. Figure 4a points out that the charge carrier density *n* increases linearly with the La concentration (except for the lowest La concentration). Such a linear correlation is expected from the observation that La is incorporated as a La^3+^ ion on Sr^2+^ sites. Thus, La can act as a donor leading to n-type semiconducting behavior in the films, which has also been discussed in ref (49). Simultaneously with increasing La concentration, we observe a significant decrease of the charge carrier mobility μ from ∼2.0 to ∼0.6 cm^2^V^–1^ s^–1^ (Figure 4b), which is attributed to enhanced scattering at an increased number of ionized impurities caused by the incorporation of La^3+^ ions on Sr^2+^ sites and/or a saturation effect.^5,50,51^ As the resistivity is related to the charge carrier mobility μ and *n* by ρ = *ne*μ, it decreases continuously with increasing La concentration (Figure 4c).

**Figure 5:**
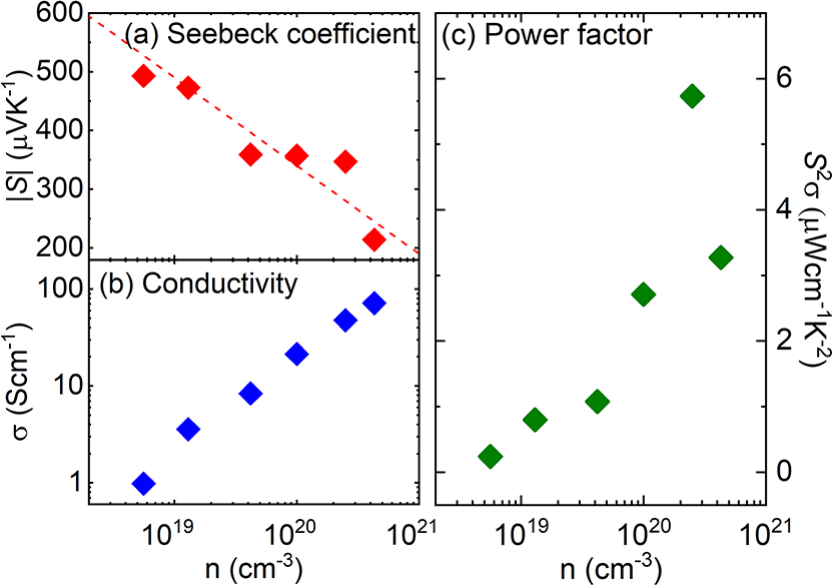
(a) Absolute value of the Seebeck coefficient |*S*|, (b) conductivity σ, and (c) power factor *S*^2^σ as a function of the charge carrier density *n*.

In order to determine the power factor as a function of *n*, we calculated the conductivity σ as a function of *n* from the data shown in Figure 4. Figure 5b reveals, as expected, a significant increase with the charge carrier density *n*. However, the conductivity σ (or resistivity ρ ∼ 1/σ) is slightly lower (higher) than corresponding values published in the literature for MBE-grown films,^12,50^ which is attributed to a slightly lower charge carrier mobility in our MOVPE films.

In the next step, the Seebeck coefficient *S* was specified for all La-doped films and is presented in Figure 5a as a function of the charge carrier concentration *n*. Our results show that *S* is negative, indicating electrons as major charge carriers. |*S*| decreases from 490 to 214 μV/K with increasing charge carrier concentration from 5.7 × 10^18^ to 4.3 × 10^20^ cm^–3^. According to refs (12, 52), the relationship of the Seebeck coefficient and the charge carrier density can be described as a straight line (see dotted line in Figure 5a):
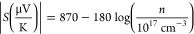


This is similar to what has been observed for other semiconductors.^12^ The absolute values for *S* for the MOVPE films are slightly smaller than those for MBE grown films^12^ and single crystals,^5^ which is attributed to the lower charge carrier mobility in our MOVPE films.

From the conductivity σ and the Seebeck coefficient *S*, the power factor *S*^2^σ was calculated (see Figure 5c). As expected, the power factor passes through a maximum, which is reached at *n* = 2.3 × 10^20^ cm^–3^ with *S*^2^σ = 5.7 μWcm^–1^ K^–2^. The evolution of the power factor agrees with literature data for MBE films^12^ and single-crystals.^5^ But as for the conductivity, the values are moderately lower, which again can be attributed to the lower carrier mobility.

Our results indicate that the introduction of La in the gas phase under MOVPE conditions leads to the incorporation of La in the perovskite structure without a significant formation of a foreign phase and to n-type semiconducting electrical behavior. Several substitution schemes for La substitution in SrTiO_3_ are described in ref (49). Due to the observed n-type conductivity of the films and the high oxygen partial pressure during deposition (see above), only the A-site substitution – La^3+^ ions on Sr^2+^ sites – is considered, which requires a charge compensation mechanism in order to achieve charge neutrality in the films. One possibility is given by the reduction of Ti^4+^ to Ti^3+^, as it is observed in Nb-doped SrTiO_3_ films grown by MBE.^46,47,53^ As previously mentioned, here no evidence of Ti^3+^ states is found, which in the first instance might be explained by a lack of sensitivity in our XPS measurements due to the low dopant concentration of the target samples, as indicated by Chambers and coworkers for films grown by oxygen plasma-assisted MBE.^47^ However, the differences between chemical (e.g., MOVPE) and physical (e.g., MBE) methods in terms of partial pressures, gas flux, deposition ratios, etc., may open diverse chemical paths to the oxide growth that may slightly influence the local chemical environment via the formation of local lattice distortions or nonstoichiometric films.

An alternative mechanism to ensure charge neutrality, especially in the case of an oxygen-rich atmosphere, is facilitated by the incorporation of additional oxygen beyond the stoichiometric composition. One possibility to accommodate the extra oxygen is the formation of a planar SrO intermediate layer between two perovskite units, comparable to the Ruddlesden–Popper (RP) phases.^54,55,59^ The formation of such SrO interlayers causes the simultaneous creation of Sr vacancies in the perovskite units. Such Sr deficient perovskite structures can exist even with a Sr deficiency of up to 10–20% in MOVPE films.^23^ Thus, the resulting composition of the La-doped films can then be described with (La_*x*_Sr_1–1.5*x*_TiO_3_)_2_·(SrO)_*x*._^56,57^ The accumulation of such ordered RP-like defect structures leads not only to ionized scattering defects but also to the formation of vertical antiphase grain boundaries,^60^ which are assumed to reduce the charge carrier mobility in lateral transport. Furthermore, increasing surface roughness due to island formation (see Figure 2b–d) and slight degradation of structural order caused by structural inhomogeneities and off-stoichiometry (indicated by a small increase of vertical lattice parameter) point to this compensation mechanism. This is assumed to be different from SrTiO_3_ thin films grown by MBE where the oxygen partial pressure is, typically, lower by 6–7 orders of magnitude.^19^

Electronic compensation and n-type conductivity are provided by the substitution of La^3+^ on Sr^2+^ sites without the formation of Sr vacancies.^49,58,59^ However, under the used deposition conditions (especially the high oxygen partial pressure), it is assumed that this only occurs at higher La concentrations in the gas phase, which might explain that more La ions are incorporated in the SrTiO_3_ thin films than are electrically active and the lower charge carrier mobility observed in our MOVPE films compared to MBE films.

In principle, charge carrier compensation would also be feasible through the formation of La_2_Ti_2_O_7_ precipitates as a foreign phase, but this interpretation does not fit to the XPS data and electrical conductivity of the films described above and is therefore ruled out.

In order to improve the electrical properties of the films, we note that a detailed analysis of the chemical and structural defects could be done by transmission electron microscopy measurements, which would, in turn, allow a fine-tuning of the deposition parameters, especially of the gas phase composition. This will be the topic of future investigations.

## Conclusions

La-doped SrTiO_3_ thin films with high structural quality were epitaxially grown on SrTiO_3_ substrates by the liquid-delivery spin MOVPE method. Careful thermal analysis of the metal–organic precursors Sr(tmhd)_2_-tetraglyme, Ti(O^i^P_r_)_2_(tmhd)_2_, and La(tmhd)_3_ provides the deposition of thin films with precisely controlled charge carrier density between *n* = 5.7 × 10^18^ and 4.3 × 10^20^ cm^–3^. This allows the determination of the electrical conductivity and the Seebeck coefficient as a function of the charge carrier concentration *n*, which reveals an optimum power factor of 5.7 μWcm^–1^ K^–2^ at *n* = 2.3 × 10^20^ cm^–3^. The lower values of the power factor compared to those obtained for MBE films are tentatively attributed to the formation of RP-like defects correlated to the formation of vertical phase boundaries and ionized scattering defects, which may trap charge carriers. Our results verify the good quality of our MOVPE-grown films and their high potential for thermoelectric applications.

## Supporting Information Available

Additional XRD data, simulations, and reciprocal space maps (PDF)
