# Supplementary material for: Potential of La-Doped SrTiO3 Thin Films Grown by Metal–Organic Vapor Phase Epitaxy for Thermoelectric Applications
Source: Cryst Growth Des. 2023 Mar 16;23(4):2522–30. doi: 10.1021/acs.cgd.2c01438 (PMC10101556; doi:10.1021/acs.cgd.2c01438)
Supplement: Supplementary file 1 — cg2c01438_si_001.pdf [file cg2c01438_si_001.pdf]

SUPPORTING INFORMATION

Potential of La-doped $SrTiO_3$ thin films grown by metal-organic vapor phase epitaxy for thermoelectric applications

Aykut Baki[1,‡], Mohamed Abdeldayem[1,‡], Carlos Morales[2], Jan Ingo Flege[2], Detlef Klimm[1], Oliver Bierwagen[3] and Jutta Schwarzkopf[1,]*

[1]Leibniz-Institut für Kristallzüchtung, Max-Born-Straße 2, 12489 Berlin, Germany

[2]Brandenburgische Technische Universität Cottbus-Senftenberg, FG Angewandte Physik und Halbleiterspektroskopie, Konrad-Zuse-Straße 1, 03046 Cottbus, Germany

[3]Paul -Drude-Institut für Festkörperelektronik, Hausvogteiplatz 5-7, 10117 Berlin, Germany

Post-annealing

As-grown $SrTiO_3$ thin films were annealed in pure oxygen flow at 800˚C for 1 h. Figure S1 reveals that both position and shape of the film contribution have not significantly changed

after post-annealing, no additional peak is observed, only thickness oscillations are slightly more pronounced. Therefore, we conclude that the films do not contain a significant amount of oxygen vacancies.

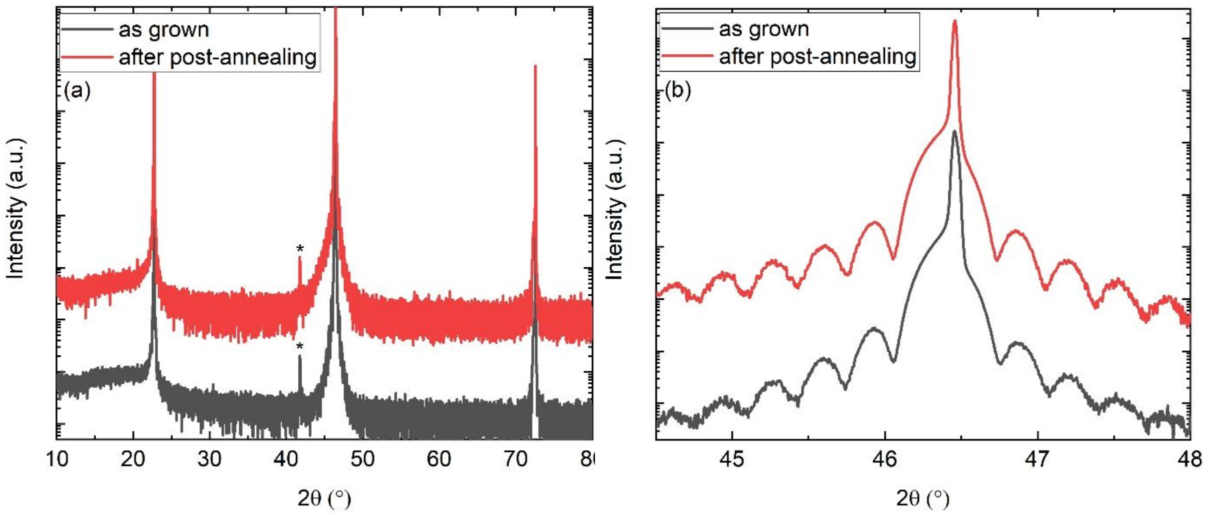

Figure S1: (a) Long-range θ/2θ HRXRD scans of the as-grown La-doped $SrTiO_3$ film (c(La) = 0.19 mM) before (black curve) and after post-annealing process, (b) same measurement with higher magnitude in the vicinity of the (002) $SrTiO_3$ Bragg peak. The peak at 41.79° (marked by an asterisk) corresponds to the substrate peak caused by the Cu Kβ line.

Coherent film growth

Reciprocal space maps (RSM) have been recorded for the undoped as well as La-doped $SrTiO_3$ thin films. Since the vertical lattice parameter of the films is only slightly larger than that of the $SrTiO_3$ substrate (see Fig. S1b), film and substrate contributions appear almost at the same q_z value. This is shown for the undoped film (Fig. S2(a)) and exemplarily for two different La concentrations (Fig. S2(b) and (c)). Also, the in-plane lattice parameters of films and the $SrTiO_3$ substrates match within the resolution limit which indicates coherent film growth. For the La-doped films, the background is slightly enhanced compared to the undoped film.

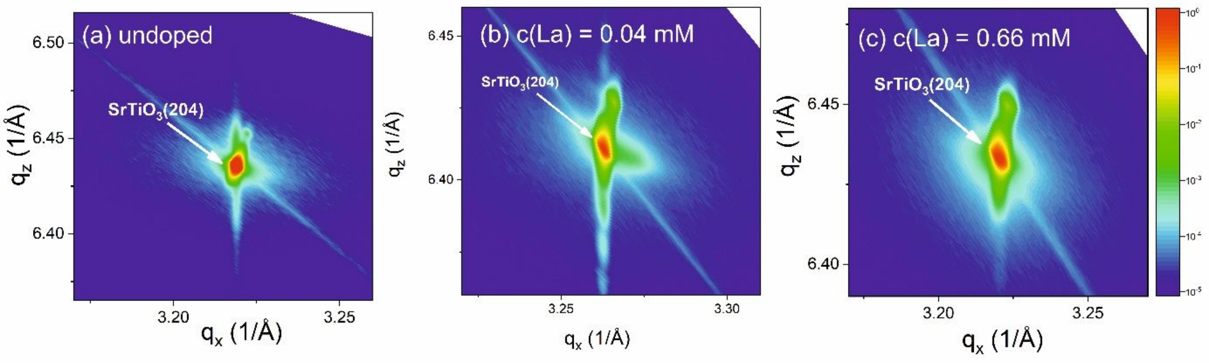

Figure S2: Reciprocal space maps in the vicinity of the (204) $SrTiO_3$ Bragg reflection of (a) the undoped $SrTiO_3$ film as well as of the La-doped $SrTiO_3$ films with (b) c(La) = 0.04 mM and (c) c(La) = 0.66 mM.

Evaluation of the XRD measurements

Simulations of the XRD scans were performed by the software RCRefSimW (Version 1.08) to evaluate film thickness and peak position. With the simplest approach of a homogeneous film coherently grown on a substrate no decent match between simulation and measurements curves could be achieved, because the simulation software does not take into account an elevated background due to dislocations and/or vacancies. Furthermore, it was also only possible to reproduce the position of the film Bragg reflection and the thickness fringes for the undoped film by the use of a single film component (see Fig. 3a). Whereas for the La-doped films, at least 2 – 3 components were always necessary to achieve a sufficiently good fit of the measured data. However, the accuracy of the results with regard to the vertical lattice parameter (from the angular position of the film peak) and the film thickness (from the distance of the thickness oscillations) could not been significantly improved with application of more than three sublayers for the film. Therefore, the maximum number of sublayers in the simulations was three.

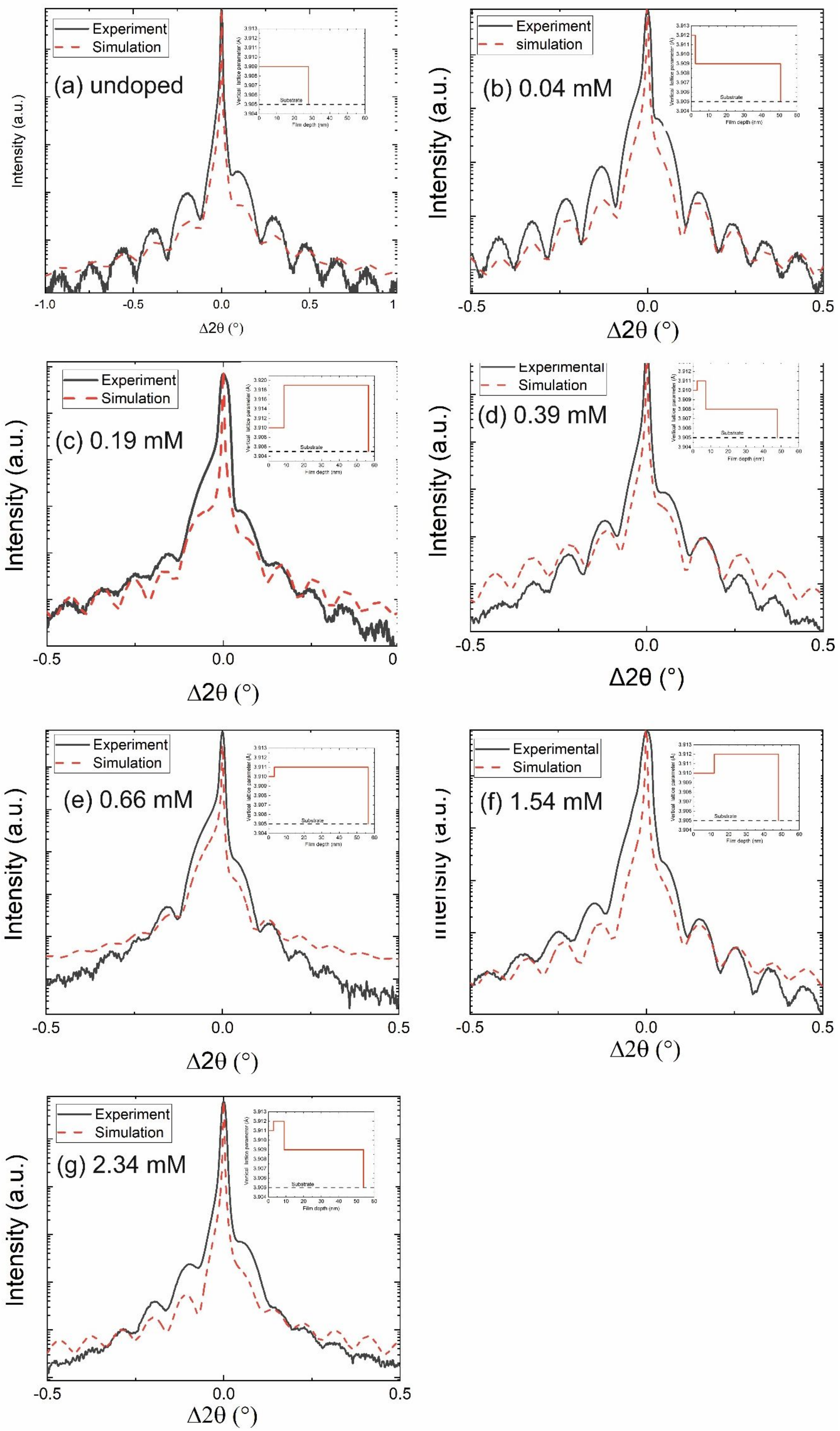

Experiment
Simulation
(a) undoped
Intensity (a.u.)
Δ2θ (°)
Experiment
simulation
(b) 0.04 mM
Intensity (a.u.)
Δ2θ (°)
Experiment
Simulation
(c) 0.19 mM
Intensity (a.u.)
Δ2θ (°)
Experimental
Simulation
(d) 0.39 mM
Intensity (a.u.)
Δ2θ (°)
Experiment
Simulation
(e) 0.66 mM
Intensity (a.u.)
Δ2θ (°)
Experimental
Simulation
(f) 1.54 mM
Δ2θ (°)
Experiment
Simulation
(g) 2.34 mM
Intensity (a.u.)
Δ2θ (°)
Substrate
Film depth (nm)

Figure S3: Measured and simulated θ/2θ HRXRD scans for the (a) undoped (c(La) = 0.0 mM) and La-doped films with (b) c(La) = 0.04 mM, (c) c(La) = 0.19 mM, (d) c(La) = 0.39 mM, (e) c(La) = 0.66 mM, (f) c(La) = 1.54 mM, and (g) c(La) = 2.34 mM.

Electrical properties

We investigated a 30nm-thick, undoped $SrTiO_3$ reference film grown under similar conditions as those discussed in the manuscript as well as an as-purchased, undoped $SrTiO_3$ substrate by Hg-CV measurement (method and setup described in Ref. S1). This method would indicate the depletion layer thickness under which a potential carrier system exists.

The measured capacitance of 3.2 pF for both, substrate and reference layers, is largely independent of DC bias and frequency confirms the absence of mobile carriers. (This capacitance would correspond to an unphysically large depletion layer thickness of ~100 μm assuming a relative dielectric constant of 200 for $SrTiO_3$). Current-voltage measurements -10V to 10V of the reference layer using the same Hg-probe also indicated insulating behavior ($R > 10^{12}$ Ω). Thus, we can safely exclude a parallel conductivity in the $SrTiO_3$ substrate under the discussed La-doped $SrTiO_3$ layers. The absence of a carrier system in the substrate is also in agreement with the less reducing growth conditions in our MOVPE (T_{growth} = 710°C,

$p(O_2)$ = 15 mbar) than those that lead to substrate reduction in Ref. [S2] with T_{growth} = 750˚C, $p(O_2) < 10^{-3}$ mbar.

S1 Pfützenreuter, D.; Kim, S.; Cho, H.; Bierwagen, O.; Zupancic M.; Albrecht, M.; Char, K.; Schwarzkopf, J. Confinement of Electrons at the $LaInO_3/BaSnO_3$ Heterointerface. *Adv. Mater. Interfaces 9, 2201279 (2022)*

S2 Herranz, G.; Basletić, M.; Bibes, M.; Carrétéro, C.; Tafra, E.; Jacquet, E.; Bouzehouan, K.; Deranlot, C.; Hamzić, A.; Broto, J.-M.; Barthélémy, A.; Fert, A. High Mobility in $LaAlO_3/SrTiO_3$ Heterostructures: Origin, Dimensionality, and Perspectives. *Phys. Rev. Lett. 98, 216803 (2007)*
